# Supplementary material for: Systematic Review on Optical Diagnosis of Early Gastrointestinal Neoplasia
Source: J Clin Med. 2021 Jun 25;10(13):2794. doi: 10.3390/jcm10132794 (PMC8269336; doi:10.3390/jcm10132794)
Supplement: Supplementary file 1 [file jcm-10-02794-s001.zip › jcm-1262878-supplementary.pdf]

**Supplementary Table S1. Systematization of search terms.** The search terms were subdivided according to the PICOS scheme

| Category | Search terms |
|---|---|
| **Population (P)** | Gastrointestinal cancer, characterization |
| **Intervention (I)** | Gastrointestinal endoscopy,<br>image enhanced endoscopy, magnification, Narrow Band Imaging / methods*,<br>image Interpretation, computer-Assisted / methods<br>precancerous Conditions / diagnostic imaging, Adenocarcinoma / diagnostic imaging |
| **Comparisons (C)** | Observer Variation, Predictive Value of Tests,<br>reproducibility of Results |
| **Outcomes (O)** | Endpoint, outcome, criteria |
| **Study designs** | Prospective, meta-analysis |

**Supplementary Table S2. Systematic literature search in MEDLINE on the primary research question "With what statistical accuracy can GN be characterized visually-endoscopically?".** The numbers behind the diamond symbol refer to the steps in the search history. Query on 05/27/2021.

| Aspects of the search | Aspect 1 | Aspect 2 | Aspect 3 | Other aspects, containment/filter | Number of results | Results after critical review of abstracts (incl. references) |
|---|---|---|---|---|---|---|
| **MeSH-term(s)** | **#1**: Early Detection of Cancer (Cancer Early Detection, Cancer Early Diagnosis) | ("diagnosis"[Mesh] OR "diagnostic imaging"[Mesh] OR "pathology"[Mesh]) | "endoscopy, gastrointestinal"[ MeSH Terms] | | 4.183 | |
| **Text words** | **#2**: Early Cancer*[tw] AND detect*[tw] | | gastrointest*[tw] AND endoscop*[tw] | | 114 | |
| **Final result** | | **Search #1 OR #12 *** | | | 254 | 21 |

* (("search"[All Fields] OR "searched"[All Fields] OR "searches"[All Fields] OR "searching"[All Fields] OR "searchs"[All Fields]) AND (("early detection of cancer"[MeSH Terms] OR ("Early"[All Fields] AND "detection"[All Fields] AND "cancer"[All Fields]) OR "early detection of cancer"[All Fields]) AND (("early detection of cancer"[MeSH Terms] OR ("Early"[All Fields] AND "detection"[All Fields] AND "cancer"[All Fields]) OR "early detection of cancer"[All Fields] OR ("cancer"[All Fields] AND "Early"[All Fields] AND "detection"[All Fields]) OR "cancer early detection"[All Fields]) AND ("early detection of cancer"[MeSH Terms] OR ("Early"[All Fields] AND "detection"[All Fields] AND "cancer"[All Fields]) OR "early detection of cancer"[All Fields] OR ("cancer"[All Fields] AND "Early"[All Fields] AND "diagnosis"[All Fields]) OR "cancer early diagnosis"[All Fields])) AND ("diagnosis"[MeSH Terms] OR "diagnostic imaging"[MeSH Terms] OR "pathology"[MeSH Terms]) AND "endoscopy, gastrointestinal"[MeSH Terms])) OR ("early cancer*"[Text Word] AND "detect*"[Text Word] AND ("gastrointest*"[Text Word] AND "endoscop*"[Text Word]))
